# Density-Gradient Mediated Band Extraction of Leukocytes from Whole Blood Using Centrifugo-Pneumatic Siphon Valving on Centrifugal Microfluidic Discs

**DOI:** 10.1371/journal.pone.0155545

**Published:** 2016-05-11

**Authors:** David J. Kinahan, Sinéad M. Kearney, Niamh A. Kilcawley, Philip L. Early, Macdara T. Glynn, Jens Ducrée

**Affiliations:** 1 School of Physical Sciences, Dublin City University, Glasnevin, Dublin 9, Ireland; 2 Biomedical Diagnostics Institute, Dublin City University, Glasnevin, Dublin 9, Ireland; Chang Gung University, TAIWAN

## Abstract

Here we present retrieval of Peripheral Blood Mononuclear Cells by density-gradient medium based centrifugation for subsequent analysis of the leukocytes on an integrated microfluidic “Lab-on-a-Disc” cartridge. Isolation of white blood cells constitutes a critical sample preparation step for many bioassays. Centrifugo-pneumatic siphon valves are particularly suited for blood processing as they function without need of surface treatment and are ‘low-pass’, i.e., holding at high centrifugation speeds and opening upon reduction of the spin rate. Both ‘hydrostatically’ and ‘hydrodynamically’ triggered centrifugo-pneumatic siphon valving schemes are presented. Firstly, the geometry of the pneumatic chamber of hydrostatically primed centrifugo-pneumatic siphon valves is optimised to enable smooth and uniform layering of blood on top of the density-gradient medium; this feature proves to be key for efficient Peripheral Blood Mononuclear Cell extraction. A theoretical analysis of hydrostatically primed valves is also presented which determines the optimum priming pressure for the individual valves. Next, ‘dual siphon’ configurations for both hydrostatically and hydrodynamically primed centrifugo-pneumatic siphon valves are introduced; here plasma and Peripheral Blood Mononuclear Cells are extracted through a distinct siphon valve. This work represents a first step towards enabling on disc multi-parameter analysis. Finally, the efficiency of Peripheral Blood Mononuclear Cells extraction in these structures is characterised using a simplified design. A microfluidic mechanism, which we termed phase switching, is identified which affects the efficiency of Peripheral Blood Mononuclear Cell extraction.

## 1. Introduction

Centrifugal microfluidic technologies [1–4] are particularly suitable for preconditioning and analysis of blood [5–12]. More recently, the commonly used centrifugal extraction of peripheral blood mononuclear cells (PBMCs) which is assisted by a density-gradient medium (DGM) [13–17] has been adapted for on-disc isolation [18–20]. In this method, the DGM is pre-loaded within a separation chamber. Blood is overlaid and, after vigorous centrifugation (typically above 400 g), red blood cells (RBCs) and granulocytes sediment to the bottom of the chamber while PBMCs and plasma are stratified on top of the DGM. While it is primarily applied for blood processing, the DGM has also been employed for bead-based immunoassays [21].

In order to avoid loss of PBMCs and thus compromise the efficiency of the extraction, the gentle and even layering of blood on top of the DGM is a crucial to the bench-top protocol. Thus, on-bench, the method can be time consuming and finicky. The Lab-on-a-Disc (LoaD) based approach can enhance reproducibility and efficiency of blood layering through the increased impact of surface tension on the micro-scale as reflected by the Weber number[22].

As all liquid volumes residing on a rotor is subject to a centrifugal field, valving techniques are pivotal for the establishment a defined, timed order of liquid handling steps. A wide variety of valves has been developed for centrifugal microfluidic systems. Amongst them are active valves [9, 23–25] which provide greater functionality; though the coordination of the various instrument-based modules tends to significantly compromise the innate conceptual simplicity of the LoaD platform. In the specific DGM case, the majority of active valves require stopping the disc for actuation. In absence of the radial centrifugal field, the stratified PBMC layer is dispersed through gravity which is directed in parallel to the axis of rotation.

For the latter, rotationally actuated valving schemes, the high centrifugal field during phase separation preclude common capillary valves [8, 26–28] as their maximum burst frequencies are severely restricted by the practical minimum feature sizes on a disc. While other burst valves[12, 29] may yield at significantly higher frequencies, flow control elements on the downstream side may not be able to tolerate the resulting high centrifugally induced pressure heads. Another approach, the rotationally independent event-triggered scheme[30, 31], is limited by the opening times of the underlying dissolvable films and thus may not be compatible with some bioassays.

Rotationally actuated siphon valves[6], which have been used for conventional plasma extraction on disc, are more suitable to the DGM method[18]. These low-pass flow-control elements remain closed at fast centrifugation and yield below a geometrically defined threshold of the spin rate. At elevated speeds of rotations, the centrifugal field suppresses capillary action which would otherwise prime the hydrophilic outlet past its crest point.

These siphon valves are particularly beneficial when placed in series with capillary burst valves to permit sequential release of liquids [32–34]. However, priming is linked to hydrophilicity of the channel walls; as the polymers commonly used for LoaD cartridges are at least slightly hydrophobic, surface coatings or surfactants need to be applied [32–34] which might interfere with the assay chemistry. Similar to the active valves mentioned above, the low / zero spin rates required for the priming of the hydrophilic channel interfere with the stratification of the layers and will thus deteriorate the extraction efficiency of the PBMC layer.

Centrifugo-pneumatic siphon valves (CPSVs) have been engineered to mitigate the need for hydrophilic surface treatments; these CPSVs transiently store energy (by centrifugally induced compression of enclosed gas) which is released upon deceleration to pump liquid through even mildly hydrophobic siphons [35–39]. As a low-pass siphon valve, CPSVs withstand the strong centrifugal field during blood separation while their opening towards low spin rates allows integration with further downstream steps towards comprehensive liquid handling automation.

In this work we present a generalised description of the CPSV scheme for blood processing with particular emphasis on comparison of so-called ‘dynamically’ and ‘hydrostatically primed’ implementations. We show how both versions enhance PBMC isolation and analyse their respective advantages and limitations. In the hydrostatic case we also improved extraction efficiency by ‘splitting’ the pneumatic chamber of the valve to stabilise the DGM layer during blood stratification.

Finally, a microfluidic effect, which we refer to as ‘phase-switching’, is identified and characterised. Here, as the DGM/plasma interface approaches the siphon outlet, the DGM is abruptly exchanged by the plasma, thus preventing part of the PBMCs from transferring to the collection chamber.

Four discs are used in this study. These discs feature (A) a single siphon, continuous pneumatic chamber hydrostatic CPSV, (B) a single siphon, split-pneumatic chamber CPSV, (C) a dual siphon, split-pneumatic CPSV and (D) a dual siphon dynamically primed / hybrid CPSV.

## 2. Generalised CPSV

In CPSVs, entrapped gas is compressed during the high-field filling process to pump liquid through the siphon channel upon reduction of the spin rate. Unlike most rotationally controlled valves, the performance of CPSVs is strongly coupled to their geometry as well as the filling dynamics of their attached reservoir. During loading, liquid must compress the trapped gas at a higher rate than the liquid can enter the reservoir such that the inner liquid meniscus remains below the crest point of the siphon.

**Fig 1:**
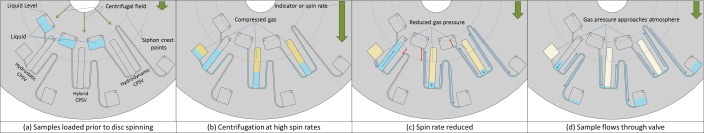
Comparison of Hydrostatic, Dynamic and Hybrid centrifugo-pneumatic siphon valves (CPSVs). Gas pressure is indicated in subfigures through the intensity of colour. (a) Liquid is loaded to the disc. (b) Upon spinning, the liquid advances into the central chamber while seeking hydrostatic equilibrium. However, the centrifugal compression of the gas volumes in the compartments enclosed by the liquid creates a counter pressure. (c, d) In the hydrostatic mechanism, the air in the closed side chamber expands upon reduction of the spin rate, so the liquid level in the open central chamber rises above the crest point of the siphon to forward the liquid into the open receiving chamber. In the hybrid CPSV, air is compressed in the closed central chamber during fast spinning. After lowering the angular frequency, the resulting decompression of air and the reduction of the centrifugal field jointly lift the liquid levels in the side arms above the crest point to empty the liquid into the open outer chamber. The operation of the dynamic CPSV follows a similar mechanism. However, the crest point of the siphon is now located above the level of the hydrostatic equilibrium; the siphon valve thus only opens upon rapid change of the spin rate so inertia propels the flow until the meniscus in the outlet channel has protruded past the liquid level in the central chamber.

We present two CPSV variants which are distinguished by the radial position of their crest points with respect to the liquid level in their central chamber prevalent at the spin rate during priming (Fig 1). In in the first embodiment, the central chamber is open to atmosphere; as the liquid level exceeds the crest point, the siphon is primed.

In the dynamically actuated CPSV, the central chamber is sealed and the equilibrium level lies below the crest point. For priming, the meniscus is pushed past the crest point through the expansion of the gas in the central chamber.

It should be noted that CPSVs reported in the literature represent a mixture of dynamically and hydrostatically primed versions, typically with a more dominant dynamic priming mechanism. For example, Zehnle *et al*. [39] modified an entirely dynamic CPSV to function as an on-disc centripetal pump. At the other end of the spectrum, Aeinehvand *et al.[37]* introduced a CPSV configuration which appears to be primarily hydrostatically primed; it employed a flexible latex membrane as a capacitive pneumatic element, thus lowering the actuation frequency of the CPSV valves.

### 2.1 Theoretical Analysis of Hydrostatic CPSVs

The compression of gas and displacement of liquid into the pneumatic chamber follows Boyle’s law ^28^ considering the geometry of the structure. A liquid volume confined by the inner and outer radii *r*_*i*_ and *r*_*o*_, respectively, and rotated at an angular frequency *ω*, experiences a centrifugal pressure head $\rho \;\bar{r}\;\Delta r\;\omega^{2}$ with the mean radial position $\bar{r}=0.5\cdot (r_{o}+r_{i})$ and liquid level difference Δ*r* = *r*_*o*_ − *r*_*i*_. This volume is also subject to the pressure $\left(\frac{V_{T}}{V_{T}-V_{D}}\right)P_{T}$ exerted by the compression of the enclosed gas which has an initial volume *V*_*T*_ at atmospheric pressure *P*_*T*_. The (incompressible) liquid volume *V*_*D*_ entering the pneumatic chamber is displaced from the sedimentation chamber.

From equilibrating the gas and the centrifugal pressures, we obtain the angular frequency

$$\omega =\sqrt{\frac{\left(\frac{V_{T}}{V_{T}-V_{D}}-1\right)P_{T}}{\rho \;\bar{r}\;\Delta r}}$$ (1)

at which the disc must rotate to displace the liquid volume *V*_*D*_ into the pneumatic chamber. The position of the (radially inward) meniscus in the sedimentation chamber *r*_*i*_ and the position of the (radially outward) meniscus in the pneumatic chamber *r*_*o*_ are coupled through the cross sections of the chambers.

The volume of displaced liquid *V*_*D*_ is directly related to the changes in meniscus positions *r*_*i*,_ and *r*_*o*_ through the known geometry of the structure. Furthermore, these values can then be used, from Eq 1, to calculate the theoretical spin rate *ω* for any given values of *V*_*D*_, *r*_*i*,_ and *r*_*o*_, required for system equilibrium.

The centrifugal force and thus hydrostatic pressure between the radially inward location of the liquid and the siphon crest reduce with the square of the spin rate. However, at the same time the liquid is displaced radially inwards above the siphon crest height. Therefore it holds that, at some frequency *ω*, the combination of liquid displacement (radially inwards of the siphon crest) and centrifugal force result in a maximum / optimum priming pressure

$$\Delta P_{p}=\rho \bar{r_{\omega }}{\Delta r_{\omega }\omega }^{2}$$ (2)

with both, $\bar{r_{\omega }}$ and Δ*r*_*ω*_, functions of *ω*.

## 3 Materials and Methods

### 3.1 Disc Manufacture

**Fig 2:**
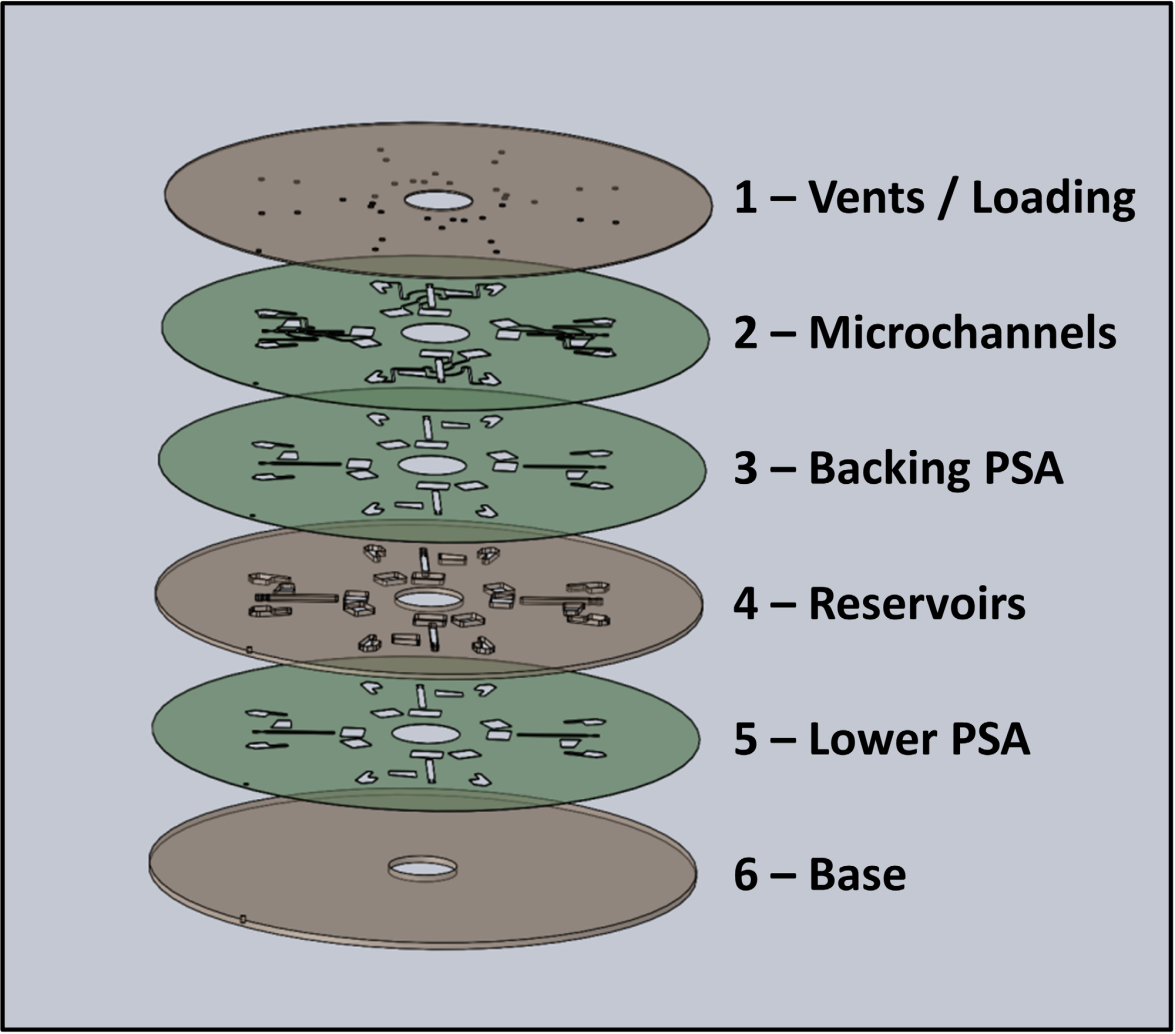
Schematic of Multi-layer discs Multi-layer discs are assembled from of 1.5-mm PMMA layers interspersed by 86-μm thin PSA films.

The discs used in this study (Fig 2) were assembled by multi-lamination of three layers of Poly(methyl methacrylate) (PMMA) and three layers of Pressure Sensitive Adhesive (PSA, 86 μm thick). Smaller features such as microchannels were created from voids cut in the microchannel layer 2 using a knife-cutter (Graphtec, Yokohama, Japan). Larger elements such as reservoirs were created from voids in the interstitial, 1.5-mm thick PMMA layer 4 with a laser cutter (Epilog Zing, USA). The top PMMA layer 1 contained loading holes and air vents while the lower PSA layer 5 and bottom PMMA layer 6 constitute the lower wall of the reservoirs. The microchannels were backed by an additional PSA layer 3 to increase hydrophilicity of the microchannels and also to improve optical contrast of images acquired. Note that, to assist with siphon priming at very low spin rates, Disc C was treated with surfactant between the capillary burst valves on the lower siphon.

### 3.2 Experimental Test Platform

The hydrodynamics on the spinning discs were characterized on an experimental test stand as previously detailed [30, 40]. In brief, a strobe light (Drelloscop 3244, Drello, Germany) and a sensitive, short-exposure time camera (Pixelfly, PCO, Germany) are synchronised with a motor (Faulhaber Minimotor SA, Switzerland) using custom electronics such that a stationary frame sequence of is acquired during rotation.

### 3.3 System Characterisation

The performance of the dual / split pneumatic chamber design was monitored using food dye in order to investigate how changing the geometry of the pneumatic chamber effected the position of the liquid heights in the disc. To simulate DGM-based processing of blood, the vents on the outlet chambers were blocked, thus preventing the siphons from priming. The chambers were filled with sufficient DI water such that, when stationary, the radially inwards liquid interface as 2 mm radially inwards of the siphon priming height (*r*_*i*_ = 23 mm). The disc was then accelerated to 60 Hz and then decelerated in steps of 5 Hz. At each frequency an image of the disc was acquired. These images were later analysed (by the software ImageJ [41]) to determine the position of the fluid interface height *r*_*i*_ in the sedimentation chamber.

### 3.4 Sample Preparation / Blood Processing / Cytometry

The study was approved by the research ethics committee of Dublin City University, with all healthy volunteers recruited with signed consent. Finger prick whole blood was used for all testing except investigation of ‘phase-switching’. The finger-prick blood extracted directly from healthy donors using 1.5-mm sterile lancets (BD Biosciences, NJ, USA) and immediately diluted at a ratio of 1:1 (100 mM PBS, pH 7.4 / 0.1 mM EDTA). The extraction efficiency (‘phase-switching’) was investigated with a venous blood sample (with EDTA at anti-coagulant).

The discs are first loaded with DGM (Ficoll Histopaque 1077, Sigma-Aldrich) so that, at the selected processing spin rate (60 Hz), the DGM liquid interface is aligned with the loading microchannel. This ensures that the liquid is layered directly onto the DGM which is a critical step for optimising the WBC isolation in the DGM method. The blood volume is selected so that the upper liquid interface of the combined blood and DGM is located below the siphon crest (~0.5 mm to 1 mm in the hydrostatically primed designs).

As the disc is accelerated, the blood flows down the loading channel and is overlaid on the Ficoll DGM. The 60-Hz spin rate is then maintained until the blood has been separated into its constituent bands. Following complete stratification, in the case of the single siphon design, the spin speed is decreased to 15 Hz in order to prime the siphons.

For investigating the extraction efficiency (‘phase-switching’), the samples were recovered from the discs and then pelleted / re-suspended repeatedly (x 3). Cell-counts were then obtained from a manual haemocytometer (Neubauer Improved Haemocytometer Slide) combined with ImageJ. Results were compared to analysis using a Hemocue WBC Diff (Hemocue) and measurement in a hospital laboratory.

## 4. Split Pneumatic Chamber

**Fig 3:**
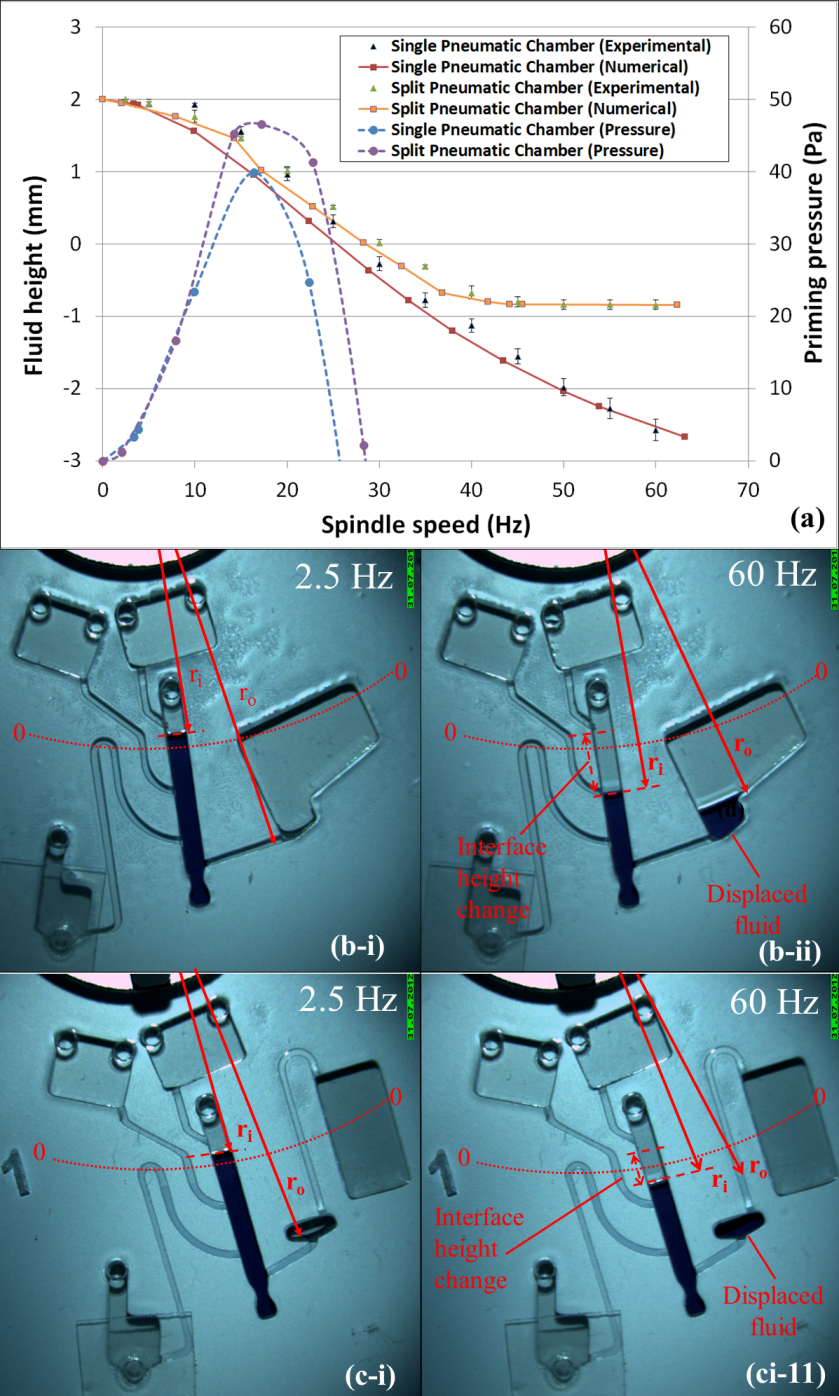
Comparison and characterisation of Discs A and B (Continuous Pneumatic Chamber vs Split Pneumatic Chamber). (a) Filling levels, relative to the datum radius (siphon crest at 25 mm), against the spin rate. Numerical modelling using Eq 1 (using volume data from 3D CAD models) is compared to experimental data (n = 4). Hydrostatic priming pressure curves are also shown (dashed lines). These curves indicate that both designs are optimally primed at about18 Hz. (b) Disc A at high (60 Hz) and low (2.5 Hz) spin rates. (c) Disc B at high (60 Hz) and low (2.5 Hz) spin rates. Note the dependence of the location of the liquid interfaces on the geometry of the pneumatic chamber. Liquid interfaces in Disc B (split pneumatic chamber) approach a stable configuration above 40 Hz. Note also that at 2.5 Hz the priming pressure is insufficient to overcome the capillary pressure in the slightly hydrophobic siphon channel.

**Fig 4:**
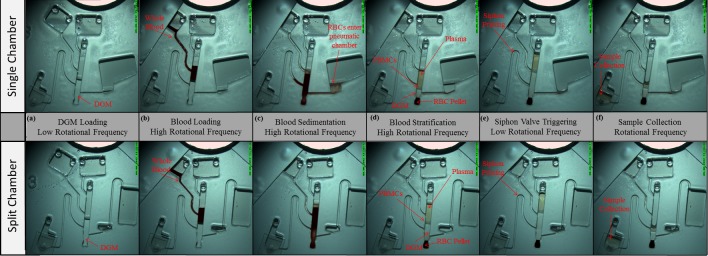
Comparison of blood centrifugation in configurations with continuous (Disc A) and split pneumatic chambers (Disc B). Note that only in the single chamber design (top) RBCs enter the pneumatic chamber due to the increased liquid displacement (c). (d) Note the different PBMC locations in the two designs above and below the siphon outlet. See ESI S1 Movie showing blood processing in Disc B.

In order to test the efficacy of using a CPSV to extract WBCs from whole blood, initially a hydrostatically primed CPSV was developed which used a continuous pneumatic chamber. However, a number of issues with this configuration were identified. In the first case, during blood loading, the increased hydrostatic pressure imposed by the additional blood displaced the PBMC layer radially outwards and excessive DGM into the pneumatic chamber (Fig 3). In some cases, RBCs were also pushed into the pneumatic chamber during this step (Fig 4).

For controlling the interface layer during loading, and thus to ensure gentle overlay of whole blood, it was identified that changing the shape of the pneumatic chamber would prevent, above a critical frequency, excessive displacement of the DGM in the system. This was achieved by splitting the pneumatic chamber into two compartments; one section designed to fill fully with DGM and the other serving to provide sufficient ballast to enable the valve to function at the desired spin rates. These two chambers are connected via a microchannel which, extending radially inwards of the sample and DGM provides a pneumatic communication while preventing a liquid transfer. Effectively, the reduced aspect ratio in the connecting microchannel (versus a wider reservoir) results, with an increase in *ω*, in a rapid change in *r*_*o*_ for a small change in *r*_*i*_. This way the system approaches an equilibrium state once the DGM enters the connecting microchannel.

The characterisation of this architecture with food dye (Fig 3) showed good agreement with numerical modelling. Additionally it was identified that, as the ‘priming pressure’ between the radially inward liquid interface and the siphon crest is dependent on both, the spin rate and the distance between the liquid interface and the crest, an optimum priming frequency can be found for each hydrostatic siphon. For the valves modelled in Fig 3, this optimum was found to be approximately 15 Hz.

In general, the use of a split pneumatic chamber offers a significant advantage towards blood processing. The stabilisation of the interface during the introduction of additional blood permits a very controlled layering of blood, which is an important aspect for efficient DGM centrifugation. Additionally, as the pneumatic chamber is pre-filled with DGM before sedimentation of RBCs initiates, minimal additional DGM liquid is displaced into the pneumatic chamber during blood layering; thus RBCs will not be displaced into the pneumatic chamber (Fig 4). In the configuration presented here, this, in turn, ensures that RBCs will not be ejected from the pneumatic chamber into the sedimentation chamber during valve priming. Finally, during blood processing, the PBMC layer is held in a defined location rather than being displaced downwards (to the extent it can often be displaced below the outlet micro-channel).

The dual-chamber design offers further advantages. In the context of of microfluidic integration, increasing the volume of the secondary chamber without changing the first, smaller chamber allows the total volume of the pneumatic chamber to be increased (thereby enabling liquid loading and valve actuation at lower centrifugal force) without changing the geometry of the sedimentation chamber or the displacement of liquid in the system. Moreover, the secondary chamber can be located at an arbitrary distance from the sedimentation chamber, thus making best use of valuable disc space.

## 5. Dual Siphon Configuration

### 5.1 Hydrostatic Configuration

**Fig 5:**
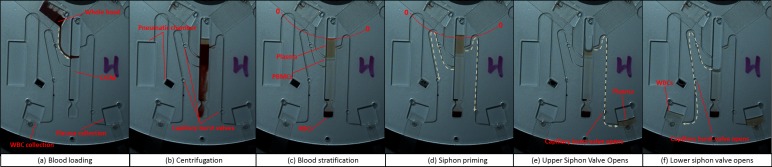
WBC Isolation using a dual siphon, split pneumatic chamber CPSV (Disc C). (a) The disc is loaded with DGM as the whole blood is introduced during disc acceleration. (b) RBCs sediment. Note that the siphons have a number of capillary burst valves. The upper capillary valve on the lower siphon prevents the DGM pre-priming siphon while the disc is stopped for blood loading. (c) Stratified blood in the chamber. Note that the plasma remains below the siphon crest points. (d) The disc is decelerated and the bulk liquid is displaced radially inwards and the siphon prime. The siphon priming is halted by the capillary burst valves. The siphons must be primed at a lower frequency (~2.5 Hz) than the nominal frequency (~15 Hz) to prevent the capillary valves from bursting early or out of sequence. However, due to the low hydrostatic priming pressure at this spin rate, the crest of the lower siphon required treatment using a surfactant to achieve reliable priming. The upper siphon was not treated. (e) The spin rate is increased and the burst valve of the upper siphon capillary is opened, thus removing the plasma to the collection chamber. (f) The spin rate is increased further and the lower siphon valve opens for removing the WBCs (with some plasma and DGM) to the WBC collection chamber. See ESI S1 Movie showing blood processing in Disc C.

The basic split-pneumatic chamber CPSV concept was further developed to present a ‘dual siphon’ design. In this case, an upper siphon removed excess plasma while a lower siphon, located below the PBMC layer, extracts the WBCs (Fig 5). This configuration has been designed with capillary burst valves which ensure that, while both siphons prime simultaneously when the spin rate is reduced, the opening of the upper siphon is followed by the second, lower siphon. This allows greater accuracy in separation of plasma from PBMCs. Additionally, the actuation of two siphons using a single pneumatic chamber permits more efficient use of precious disc real-estate compared to sequential configurations[36].

### 5.2 Dynamic / Hybrid Configuration

**Fig 6:**
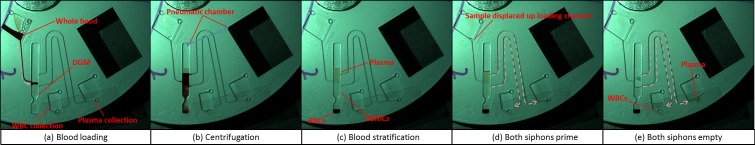
WBC isolation using a dynamically CPSV (Disc D). (a) The disc loaded with DGM while the whole blood is introduced during disc acceleration. (b) RBCs sediment to the bottom. Note that the pneumatic chamber is extended by channel (lower level) indicated in a blue dash. This large pneumatic chamber is required to ensure that the valves function at the volumes processed. (c) Stratified blood in the chamber. (d) The spin rate is decreased and both siphons are simultaneously primed. Note that the siphon crests are located radially inwards of the bulk liquid and the liquid displaced radially inwards along the loading channel. (e) The spin rate is increased and both siphons empty. See ESI S1 Movie showing blood processing in Disc D.

A hybrid / dynamically primed dual-siphon scheme is also demonstrated. In this case, the bulk of the sample liquid is located in the pneumatic chamber. The base configuration was connected via a microchannel to a larger pneumatic chamber, located away from the CPSV valve, to permit processing of blood volumes at practical spin frequencies of the disc (Fig 6).

## 6. Extraction Efficiency and Phase Switching

**Fig 7:**
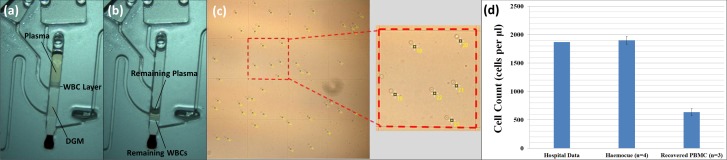
**Phase Switching Data and Quantitative Results** (a-b) highlight ‘phase-switching’. This trait occurs where the system switches from drawing one phase, the DGM, to the other phase, plasma, while leaving a significant number of PBMCs within the main sedimentation chamber (c) and image from the haemocytometer showing mononuclear leukocytes enumeration (d) comparison of mononuclear leukocytes extracted from the single pneumatic chamber (Disc A) to a whole blood count (hospital laboratory) and using a HemoCue™.

In order demonstrate the efficacy of PBMC extraction, cells extracted from 18 μl whole blood were recovered from Disc B, re-suspended in buffer and then enumerated using a haemocytometer (Fig 7C and 7D).

However, as can be seen in Fig 7A and 7B, it appears that a quantity of PBMCs remain in the blood processing chamber after the siphon has discharged. This is particularly evident in the movies provided in the ESI. Qualitatively, it appears that the siphon first draws on the DGM phase and then abruptly changes to drawing on the plasma phase. Samples recovered from Disc B and enumerated reveal a rather low PBMC extraction efficiency of just 34% compared with cell counts using a hospital laboratory and a Hemocue WBC Diff (Hemocue™).

One potential solution to this ‘phase switching’ is to locate the inlet of the extraction siphon in a funnel-like constriction. However, this will most likely result in a ‘Reverse Boycott Effect’ where congestion and clogging in the channel increased the time for blood sedimentation from the order of a few minutes to hours.

## 7. General Discussion

In this work we present PBMC extraction with hydrostatic and hydrodynamic CPSV valves. In the hydrostatic case, the system uses a ‘gentler’ priming mechanism and, in general, could function at moderate acceleration and deceleration ramps. As the priming is driven by hydrostatic pressure, a dual-siphon system can have integrated capillary burst valves to control the order at which the siphons empty. However, it must also be noted that this approach can have reliability issues; in some cases (~50%) where the structures were untreated only one of the siphons will prime; thus for our work here we applied a surfactant to one of the siphons.

For the hydrostatically primed valves, the use of a ‘split pneumatic chamber’ also offers certain benefits. Alongside increased stability of liquid interfaces at high spin rates, the split-pneumatic chamber permits the tailoring of the burst frequencies by altering the volume of the ‘ballast’ chamber, without changing any (other) critical dimensions of the microfluidic structure. Similarly, this also allows more efficient use of disc real estate as the ‘ballast’ chamber can be located anywhere on the disc.

A significant plus of the dynamically primed valving scheme is its enhanced reliability compared to the hydrostatic case. Another advantage is that, as bulk of the liquid is not moved between two chambers during sedimentation, the DGM / blood interface is stationary during processing. Yet, this approach also entailed a number of challenges. Primarily, this approach requires quite aggressive disc acceleration rates to prevent premature priming of the siphons. Similarly, a relatively large pneumatic chamber is needed to process a relatively small blood volume at the spin rates (60 Hz) used in this study. This requirement could be moderated by making the blood processing chamber wider; however it is postulated that this would increase the adverse impact of the ‘phase switching’ trait identified here.

## 8. Conclusions and Outlook

In this study we present a repertoire of CPSV configurations which are optimised for blood processing. We demonstrate how the geometry of the pneumatic chamber in a hydrostatic CPSV can significantly decrease the liquid displacement which occurs in the valve. We also identify that, for each hydrostatic CPSV, an optimal siphon priming frequency can be found.

Additionally, we present a significant advancement where we show, for both hydrostatic and dynamically primed siphons, the priming of two siphons using a single pneumatic chamber. This configuration is particularly applicable to the DGM technique as it permits the separation of whole blood into its constituents plasma, PBMCs and RBCs. Finally, we identify a microfluidic trait, called ‘phase-switching’ which reduces the extraction efficiency of these structures.

It should be also noted that this platform was characterised using a white-light, haemocytometer based WBC count. This method does not reflect potential RBC and neutrophil contamination. Similarly, the extraction efficiency falls below that of current state-of-the-art techniques. However, with optimisation, the platform offers potential to form the basis of the initial blood processing step for a wide variety of applications such as CTC detection [42], CD4+ enumeration for HIV diagnostics [43, 44] and white blood cell differential counting [45, 46].

## Supporting Information

S1 Movie Supporting Movie. Blood processing in Discs B, C and D at 8x normal time
(MP4)
